# How Important is Parental Education for Child Nutrition?

**DOI:** 10.1016/j.worlddev.2017.02.007

**Published:** 2017-06

**Authors:** Harold Alderman, Derek D. Headey

**Affiliations:** The International Food Policy Research Institute, USA

**Keywords:** parental education, schooling quality, undernutrition, stunting

## Abstract

Existing evidence on the impacts of parental education on child nutrition is plagued by both internal and external validity concerns. In this paper we try to address these concerns through a novel econometric analysis of 376,992 preschool children from 56 developing countries. We compare a naïve least square model to specifications that include cluster fixed effects and cohort-based educational rankings to reduce biases from omitted variables before gauging sensitivity to sub-samples and exploring potential explanations of education-nutrition linkages. We find that the estimated nutritional returns to parental education are: (a) substantially reduced in models that include fixed effects and cohort rankings; (b) larger for mothers than for fathers; (c) generally increasing, and minimal for primary education; (d) increasing with household wealth; (e) larger in countries/regions with higher burdens of undernutrition; (f) larger in countries/regions with higher schooling quality; and (g) highly variable across country sub-samples. These results imply substantial uncertainty and variability in the returns to education, but results from the more stringent models imply that even the achievement of very ambitious education targets would only lead to modest reductions in stunting rates in high-burden countries. We speculate that education might have more impact on the nutritional status of the next generation if school curricula focused on directly improving health and nutritional knowledge of future parents.

## Highlights

• Assesses impacts of parental education on child nutrition for 56 countries.
• Conventional estimates may be substantially biased.
• Impacts are larger for mothers and larger for secondary education than primary.
• Impacts larger in countries with high stunting and more educational quality.
• Even ambitious education targets will only lead to modest reductions in stunting.

## 1 Introduction

Stunting contributes to overall child mortality (Bhutta *et al.*, 2013) and also reduces the productivity of survivors when they enter the workforce (Hoddinott, Alderman, Behrman, Haddad, & Horton, 2013). Thus, there is a strong economic as well as humanitarian rationale for improving nutrition. However, one authoritative estimate suggests that scaling up proven effective nutrition-specific interventions in the world’s most malnourished countries would only reduce stunting globally by 20% (Bhutta *et al.*, 2013). Therefore, additional actions in “nutrition-sensitive” sectors will be critical components of any global strategy to eliminate undernutrition (Ruel & Alderman, 2013). Among these, education absorbs the largest share of the development budget in low- and middle-income countries, at just over a third (IFPRI, 2014).

The scale of education investments, of course, would avail nutritional health little if the investments did not have a sizeable impact on undernutrition. But although there has been extensive research on the associations between the education of adults and the health status of the next generation (Behrman and Wolfe, 1984, Behrman and Wolfe, 1987, Desai and Alva, 1998; Duflo and Breirova, 2004; Fafchamps and Shilpi, 2014, Headey, 2013, Thomas et al., 1991), obtaining rigorous experimental evidence, which would permit stronger causal interpretation, is challenging. Impact evaluations would need to track the intergenerational effects of randomized education investments and cover a range of education levels. 1 Exploiting natural experiments is therefore a more common research strategy, though these too have limitations (Card, 2001). Perhaps unsurprisingly, then, the literature on the child health impacts of parental education is still characterized by several longstanding controversies.

The first is whether there is a threshold or minimum amount of education necessary to have measureable impacts on nutrition. This is important since many countries are now close to reaching the much lauded target of universal primary education although this progress has not yet translated to universal primary completion in many low-income settings (World Bank, 2016). Yet non-linear returns to education may emerge from many factors: primary and secondary schools might vary greatly in quality and in impacts on nutritional knowledge, labor force outcomes and marriage market outcomes; secondary schooling for girls, but not boys, might postpone childbearing; and education may complement or substitute for other factors, such as household wealth or women’s empowerment. Thus, there is value in assessing non-linear impacts of education across a heterogeneous group of developing countries.

A second related question is whether maternal education yields greater health benefits for the next generation than paternal education.^2^ Although many developing countries make extra efforts to keep girls in school, the evidence on there being greater social returns to maternal education remains controversial. Basic multivariate analysis typically suggests that, in developing countries especially, maternal education has stronger child health and nutritional associations than paternal education (Desai and Alva, 1998, King et al., 2008, Vollmer et al., 2016). It is also widely perceived that women on average wish to have fewer children than men (Ashraf, Field, & Lee 2014),^3^ and that mothers devote more resources to their children than fathers do (Yoong, Rabinovich, & Diepeveen, 2012). On this basis many international development institutions have strongly advocated investing in women’s education (Summers, 1992, King et al., 2008, World Bank, 2012), and development targets often include gender parity in education outcomes as a stated goal.^4^ However, more nuanced economic analyses argue that the least squares estimates of the effects of maternal education on child health are likely to be more biased than the corresponding estimates for paternal education in environments characterized by discrimination against women. Intuitively, in a discriminatory environment it is only mothers with innate ability or exceptional childhood circumstances (e.g. exceptionally well educated parents) that will be able to attain higher levels of education, leading to larger omitted variables bias relative to paternal education levels. Consistent with this intuition, several quasi-experimental studies find that standard multivariate analyses from observational data yield upward-biased estimates of the returns to education, particularly women’s education, relative to more experimental econometric approaches (Duflo, 2012, Breierova and Duflo, 2004, Fafchamps and Shilpi, 2014).^5^

Finally, there remains a significant knowledge gap on the question of *why* parental education matters for the health outcomes of the next generation. A small literature has examined whether formal education influences nutrition primarily by imparting literacy and numeracy skills, or whether education empowers women, or whether schooling directly exposes future parents to health information and knowledge (Glewwe, 1999, Webb and Block, 2004). Yet, to our knowledge, these various linkages have not been tested with unit-level data for a wide range of countries.

In this paper we seek to sharpen our understanding of the role of education on undernutrition by exploiting the widely used Demographic Health Surveys (MEASURE DHS, 2015). Specifically, we analyze the linkages between parental education and child health from 134 Demographic Health Surveys (DHS) for 376,992 preschool children from 56 developing countries. These data permit us to address the various knowledge gaps described above. We first address the question of endogeneity biases by comparing a simple least squares model to a model with cluster fixed effects to control for community characteristics, and finally to a model that includes a parent’s educational rank within their location-specific cohort as an additional control for unobservable ability or family characteristics, following Fafchamps and Shilpi (2014), who analyze parental education’s impact on non-nutritional health indicators for Nepalese children. We then explore issues of parameter heterogeneity by exploring whether the returns to education vary with household wealth and gender norms, national stunting rates, and a simple proxy for educational quality based on functional literacy. Finally, we use the rich array of indicators in the DHS to explore some of the possible mechanisms that might explain differences in the returns to maternal and paternal education.

The remainder of this paper is structured as follows. Section 2 reviews our data and Section 3 our methods. Section 4 presents our main results directly linking child nutrition outcomes to parental education. Section 5 explores potential explanations of our findings, and their implications for policy. Section 6 concludes.

## 2 Data

The DHS survey instrument focuses on health and basic welfare of women of reproductive age and their children, and is designed to be representative at a national level as well as at urban, rural and subnational levels. The DHS are widely regarded as high quality and are particularly advantageous for multi-country analysis because of their standardization. For this paper we merged all applicable DHS rounds across countries and rounds and standardized relevant indicators.

Summary statistics for the core indicators used in the majority of our regressions are reported in Table 1, while Table 2 reports descriptive statistics for HAZ scores and parental education for each of the five major developing regions, as classified by the World Bank.^6^

The primary outcome variable for this study is the Z score for height for age (HAZ) of children 25–59 months based on the current WHO norms available at http://www.who.int/nutgrowthdb/en/. Height for age is an indicator of cumulative nutrition and thus a measure of the stock of health that is produced, in part, by the stock of education. In order to estimate the full effects of parental education on pre-school nutrition we excluded children 0–24 months of age, which corresponds to the “first 1,000 days” of life a period over which most growth faltering takes place (Victora, de Onis, Curi Hallal, Blössner, & Shrimpton, 2009). Since parental education can influence nutrition through many postnatal as well as prenatal investments, including younger children (0–24 months) in the sample would underestimate the nutritional returns to education, because some of these returns would not have been fully actualized for very young children. For example, parental education might improve child feeding practices in the crucial 6–24 month period (a hypothesis we test below), but measuring HAZ at age 5 months would not capture this mechanism. After applying this important exclusion, we were left with a data set consisting of over 376,992 preschool children from 56 developing countries.^7^

As expected, given the DHS country selection, the mean HAZ score is a low −1.64, and 40% of our sample of children are stunted (HAZ < −2). Notably, almost 50% of our sample is from sub-Saharan Africa. In contrast, Eastern Europe and Central Asia—where child undernutrition is very low and education levels very high—has relatively few observations. Similarly, the East Asian sample contains surveys from only two countries (Cambodia and Timor-Leste) since other surveys in the region did not collect anthropometry. Thus, this sub-sample is underrepresented and excluded from some of our regional comparisons. Samples for some regions are also dominated by a few countries. Egypt—admittedly a large country with relatively high rates of stunting—accounts for almost three quarters of the Middle East and North Africa sample, and Peru accounts for just under half of the Latin America and Caribbean sample. Hence the non-representative nature of the selection of DHS countries should be borne in mind when interpreting some of the results below.

**Figure 1:**
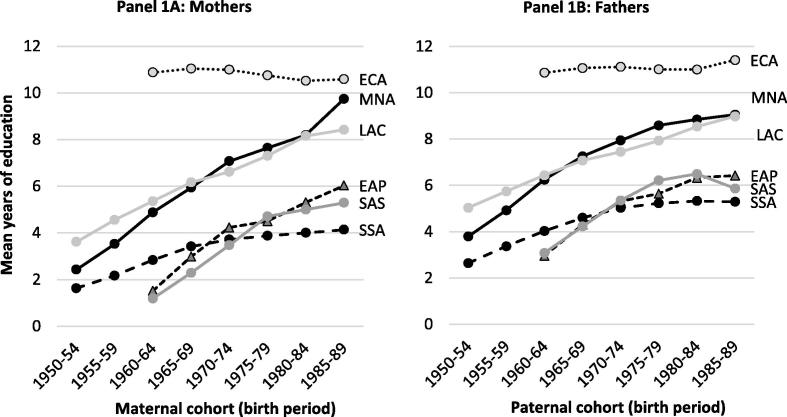
Trends in maternal and paternal education across cohorts and by region. *Source:* Authors’ estimates from DHS data. See text for details. Notes: These estimates are based on simple means by age-cohort, defined as five-year birth periods. Regional groups are World Bank classifications: ECA = Eastern Europe and Central Asia; MNA = Middle East and North Africa; LAC = Latin America and Caribbean; EAP=East Asia and Pacific; SAS = South Asia; SSA = Sub-Saharan Africa. 1950–54 and 1955–59 cohorts are missing for ECA and SAS because the surveys in these regions were more recent and did not include parents born in these periods.

The primary explanatory variable in our study is the extent of parental education, as measured by years of formal schooling.^8^ Table 1, Table 2 show tremendous variation in parental schooling, as reflected by large standard deviations in Table 1, and marked regional variations in Table 2. Unsurprisingly, education levels are easily the highest in Eastern Europe and Central Asia, where virtually all parents have at least completed primary school and gender gaps in education are relatively small. Levels of schooling attainment are also relatively high in Latin America and the Middle East and North Africa (6 to 8 years on average), but much lower in sub-Saharan Africa and South Asia (less than 6 years), where gender gaps are still quite large (1.3 years in the former and 1.6 years in the latter). Trends in parental education by age cohort also show very marked differences across regions. Figure 1 plots mean education levels by parental age cohorts for parents older than 21 years of age. Eastern Europe and Central Asia scarcely show any intergenerational expansion in education (since education levels there were already very high during the Soviet era), whereas schooling investments in Latin America, the Middle East and Northern Africa have expanded very rapidly. The speed of change in South Asia has been similarly rapid, but sub-Saharan Africa shows a very different trajectory, with rapid education gains from independence to the 1980s, after which time there was only a very modest expansion of educational attainment. This is likely related to Africa’s economic stagnation the mid-1980s to around the turn of the millennium.

**Figure 2:**
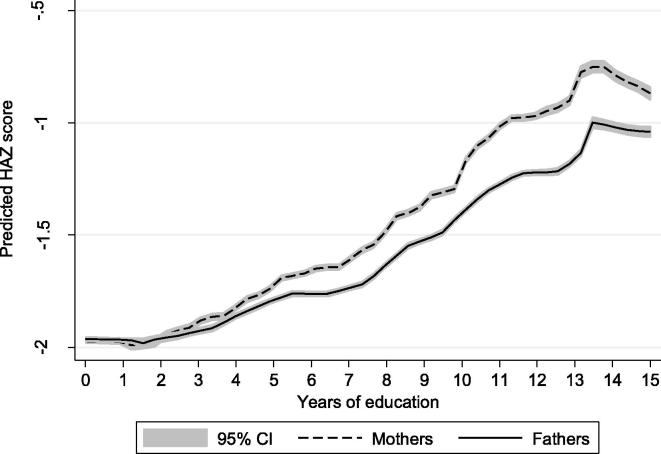
Stunting prevalence by years of maternal and paternal education. *Sources*: Authors’ estimates from the DHS rounds listed in Table 1. These are local polynomial smoothing estimates with 95% confidence intervals (CI).

Figure 2 looks at the simple bivariate association between parental education and child HAZ scores using local polynomial smoothers with 95% confidence intervals. Two facts are readily apparent from the figure. First, the slope is non-linear, being essentially flat for the first few years of education, rising gradually until about 7 years of education, rising more steeply until 13 years of education, before flattening out again. Second, the slope is noticeably steep for mother’s education. At 10 years of education, for example, there is a 0.2 standard deviation difference in mean HAZ scores between maternal and paternal education. We explore this non-linearity using a flexible approach whereby we pool years of education into 3-year brackets. This bracketing of years of education has the advantage of simplifying the reporting of our results and of broadly corresponding to basic schooling categories, such as the near completion of primary school (4–6 years), attending what is variously termed “lower secondary” or “middle school” (7–9 years), attending “upper secondary” (10–12 years) and achieving some amount of tertiary education (13 plus years).

Apart from basic location and demographic controls (for child age, mother’s age and father’s age), the other main control variable in our models is the household wealth index, which is of some importance given its strong association with both parental education and HAZ/stunting. While wealth or asset indices are now widely used in the literature (following Filmer & Pritchett, 2001), one point of note is that we mostly use country-specific wealth indices in this study, which were constructed via principal components analysis. In our regression models we also allow this variable to have country specific impacts. The reason for doing so is because different assets in different economies may have different associations with the latent variable of interest, household wealth. One exception to the country-specific wealth indices is that we use a wealth index constructed from the pooled sample of countries when conducting interaction tests between the wealth and education variables.

Finally, our supplemental results include a number of additional variables used as dependent variables to explore causal pathways between child nutrition and parental education. These are described in more detail below, but include fertility rates, maternal and child dietary diversity scores, health service utilization, and proxies for maternal decision-making/empowerment.

## 3 Methods

Underlying our analysis is a standard conceptual model with nutrition as the outcome of a health production function in which households combine food, health, and sanitation inputs to produce nutrition and other welfare outcomes under constrained resources (finances, time). The inputs obtained depend on their prices as well as the resources of the household and their preferences for investments in children. The model, however, is only implicit; it is not possible to identify the underlying demand for these inputs with the data at hand. Thus, any estimated production function is potentially biased to the degree that the level of inputs used is correlated with unobserved skills. Therefore, the study estimates a function in which the conditional demand for health in any period is dependent on time-varying demographic characteristics, non-health human capital and background characteristics, both of which are time invariant having been determined in an earlier period; assets at birth, as well as local infrastructure (Strauss & Thomas, 2007). Specifically, our most basic model takes the form:

(1) $$N_{i\text{,}j\text{,}k}=\beta^{m}E_{i\text{,}c\text{,}k}^{m}+\beta^{p}E_{i\text{,}c\text{,}k}^{p}+\beta^{h}H_{i\text{,}j\text{,}k}+\beta_{k}^{W}W_{i\text{,}c\text{,}k}.\mu_{k}+\beta_{k}^{D}D_{i\text{,}j\text{,}k}.\mu_{k}+\mu_{k}+\varepsilon_{i\text{,}j\text{,}k}$$

where *N* is child anthropometric status, ***E*** is a series of dummy variables for maternal and paternal education brackets (superscripts *m* and p*,* respectively), *H* maternal height (which might be correlated with a mother’s education), ***W*** refer to household wealth, and ***D*** refers a series of child and parental demographic characteristics (age, sex of child, rural/urban location), and *i*, *j*, and *k* respectively denote child, cluster and country identifiers. The wealth variables are interacted with country dummy variables (since they are ordinal) as are the child age dummies to allow for the relatively large cross-country differences in child growth processes (Victora *et al.*, 2009).

The coefficients on $\beta^{m}$ and $\beta^{p}$ yield information on the nutritional returns to parental education, the extent of non-linearities in these returns, and whether these returns differ significantly between mothers and fathers. However, as we noted above, there is a concern that since schooling choices are endogenous there is a risk that results linking education and subsequent outcomes may be biased (Duflo, 2012, Fafchamps and Shilpi, 2014). On the supply side, one legitimate concern is that the placement of schools could be correlated with other factors that influence nutrition, such as general levels of community development and infrastructure, thus leading to inflated estimates of the returns to education. We address this concern by including cluster fixed effects in the regressions.^9^ As is often the case, while reducing potential upward bias from positively correlated infrastructure, this step underestimates any indirect influence of community shared knowledge (Basu and Foster, 1998, Alderman et al., 2003).

On the demand side, a significant concern is that unobserved individual and family characteristics simultaneously determine both parental education and child nutrition outcomes, and hence may lead to biased estimates of the returns to education. For example, the education of a parent might reflect innate ability since more gifted children typically progress further in school or receive more investments from parents or educators, or it might reflect intergenerational family values that could influence nutrition independent of education mechanisms. To address this issue Fafchamps and Shilpi (2014) suggest that a parent’s educational rank relative to its peers will capture much of the unobservable characteristics that, if omitted, could bias conventional least squares estimates.^10^ Specifically, they measure the educational rank of a parent relative to parents of the same age cohort from the same geographical region. We do the same, measuring cohorts in 5-year increments, and using the lowest subnational region at which the DHS is representative (e.g., states, provinces or, in a few cases, districts). Moreover, we rescale this ranking indicator to vary between zero and one in each region-cohort (effectively a percentile), such that the scale of the variable has some interpretation.

Thus, the most stringent of our specification adds both cluster fixed effects ($\mu_{j}$) and educational ranks $\left(\overset{˜}{E}\right)$ to regression (1):

(2) $$N_{i\text{,}j\text{,}k}=\beta^{m}E_{i\text{,}j\text{,}k}^{m}+\beta^{p}E_{i\text{,}j\text{,}k}^{p}+\beta^{h}H_{i\text{,}j\text{,}k}+\beta_{k}^{W}W_{i\text{,}j\text{,}k}.\mu_{k}+\beta_{k}^{D}D_{i\text{,}j\text{,}k}.\mu_{k}+\mu_{k}+\mu_{j}+\beta^{\overset{˜}{m}}{\overset{˜}{E}}_{i\text{,}j\text{,}k}^{m}+\beta^{\overset{˜}{p}}{\overset{˜}{E}}_{i\text{,}j\text{,}k}^{p}+\varepsilon_{i\text{,}j\text{,}k}$$

The coefficients on $\overset{˜}{E}$ may also give some indication as to the extent of the bias, and we would naturally expect these variables to be positively correlated with nutrition. Moreover, Duflo (2012) and Fafchamps and Shilpi (2014) note that if girls are typically discriminated against in terms of educational investments, then the upward bias on maternal education might be larger than the bias associated with paternal education (more so in more discriminatory environments).^11^

We also test whether these associations with education are sensitive to the exclusion of wealth, whether they are the same across wealth rankings, and whether the pooled sample results are sensitive to sub-samples. In terms of sub-samples, we first split the sample up into high- and low-undernutrition burden countries defined by a threshold of 30% stunting, our expectation being that education likely matters more for child nutrition in higher burden countries. We then split the sample into “low-quality education” and “high-quality education” countries defined by a threshold of 50% literacy (ability to read a complete sentence) among mothers with exactly 5 years of education. Our expectation is that higher quality education translates into larger nutritional impacts, though we acknowledge the limitations of this quality measure, namely that it is primary-school specific and gender-specific (unfortunately, male literacy data are not universally collected by the DHS). In addition to these thematically defined sub-samples, we also divide the full sample into major regions, which vary in terms of both stunting rates, average education levels and education quality.

Finally, we explore whether there are significant gender differences in the estimated effects of education on some of the causal pathways linking parental education to child nutrition. To this end we replace child nutrition in Eqn. (2) with various indicators of some of the more proximate determinants of child nutrition, including fertility (children ever born), dietary diversity of mothers and children, health services utilization, and maternal decision making indicators pertaining to child and maternal health decisions. As with Eqns. (1), (2), we again test whether the parental education coefficients exhibit significant gender differences.

## 4 Results

Table 3 reports our core results for the entire sample. Regressions 1, 2 and 3 refer to HAZ score results, and regression 4 takes stunting (HAZ < −2) as the dependent variable. While columns 3 and 4 of Table 3 represent our preferred specification it is instructive to report the results of the more naïve models presented in regressions 1 and 2. Regression 1 does not control for either fixed effects or education rank relative to the parent’s age and regional cohort. In this rather naïve regression model the returns to maternal education appear especially high: a child of a mother with some tertiary education would be expected to have a child almost 0.5 standard deviations taller than a child with a mother with no education, controlling for household wealth, child and parental age. In most cases the point estimates on maternal education levels in regression 1 are twice as large as the corresponding levels for paternal education. In regression 2 we add fixed effects to the HAZ model, which controls for community characteristics that might be simultaneously correlated with both school attainment and nutrition outcomes. The coefficients on both maternal and paternal education brackets decline markedly, but more so for mothers (falling by more than half for some education brackets).^12^ Finally, regression 3 adds each parent’s education rank relative to their age cohort and subnational region. The positive signs indicate that children of those individuals who have more education than their regional and age cohorts have better nutrition, indicating a positive correlation between education rank and the unobserved characteristics such as the ability of the parents to provide health and child care. Controlling for these factors also further suppresses the apparent marginal effects associated with the main education brackets for both mothers and fathers. The highest level of maternal education now only has a marginal effect of 0.21 standard deviations, as compared to 0.52 in the most naïve model. Wald tests on the coefficients reported in regression 3 suggest that at higher levels of education the coefficients of maternal education are significantly larger than those of paternal education. Likewise there is evidence of increasing nutritional returns to maternal education, but no significant evidence of increasing returns to paternal education.

Regression 4 in Table 3 reports the results of a linear probability model for stunting (HAZ < −2), which is analogous to regression 3 with controls for community fixed effects and educational rank. The marginal effects in this specification can be interpreted as changes in stunting probabilities. Relative to the 0–3 year category, a woman with 7–9 years of education has a child who is 2.4 percentage points less likely to be stunted. That is, stunting decreases by 7% of the average over the sample with this level of education. This effect increases to a 4.8 point reduction with 10–12 years of education and 5.5 point reduction for 13-plus years of education. The analogous coefficients for paternal education are smaller: 1.9 points, 2.9 points and 3.6 points respectively.^13^ We also note that, consistent with the idea that parental education influence HAZ through postnatal channels, using the full sample of 668,000 children 0–59 months of age generally reduces the coefficients on both maternal and paternal education relative to the 25–59 month sample, often by 10% or more (results reported in Appendix Table 10).

Given the relatively modest coefficients on parental education reported in the more stringent regression model in Table 3 (regression 3), the remainder of our analysis explores the sensitivity of this more restrictive model to alternative specifications and samples.

In Table 4 we test for significant interactions between parental education and household wealth. Previous studies have investigated whether parental education might complement or substitute for socioeconomic status (Barrera, 1990, Jalan and Ravallion, 2003, Leroy et al., 2014). For example, substitution effects might entail educated women in poor households being able to make better nutritional use of limited household resources; alternatively, educated women may not be able to translate better knowledge into improved outcomes if they cannot afford to do so, such that education and wealth are complements. Table 4 tests this hypothesis using linear specifications for parental education and an asset-based poverty indicator equal to 1 if a household falls within the bottom 40% of a wealth index constructed for the entire sample as a whole. In both the HAZ model (regression 1) and the stunting model (regression 2), the sign on the “asset poor” indicator is negative and positive, respectively, and highly significant in both cases. Moreover, there is a highly significant negative interaction between maternal education and asset-poor, indicating that maternal education and household wealth are complements rather than substitutes. Indeed, the estimated effect is large: being poor erodes almost half the benefit of a given level of maternal education. For poor households there is also no significant difference between the coefficients on maternal and paternal education, although it should be noted that asset-poverty rates in households where women have 10 or more years of education are very low (5% or less). We also note that the results in Table 4 are robust to specifying an interaction between years of education and a continuous wealth index defined in terms of percentile ranking.

Appendix Table 11 also examines the sensitivity of the results in Table 3 to the exclusion of household wealth. In our core results reported Table 3 we included household wealth because inheritable wealth, in particular, might be a determinant of both parental education as well as the socioeconomic status of the household. However, since parental education also likely contributes to household wealth, it is possible that the estimates in Table 3 are lower bounds estimates of the benefits of parental education for child nutrition. Appendix Table 11 shows the expected result: the coefficients on all the education brackets increase substantially, typically in the range of 20–50%. However, the patterns of significant and coefficient magnitudes across education brackets, gender, and specification remain very similar, such that the main conclusions drawn from Table 3 are materially unchanged: the returns to education still appear to be increasing in magnitude, and higher for mothers.

**Figure A1:**
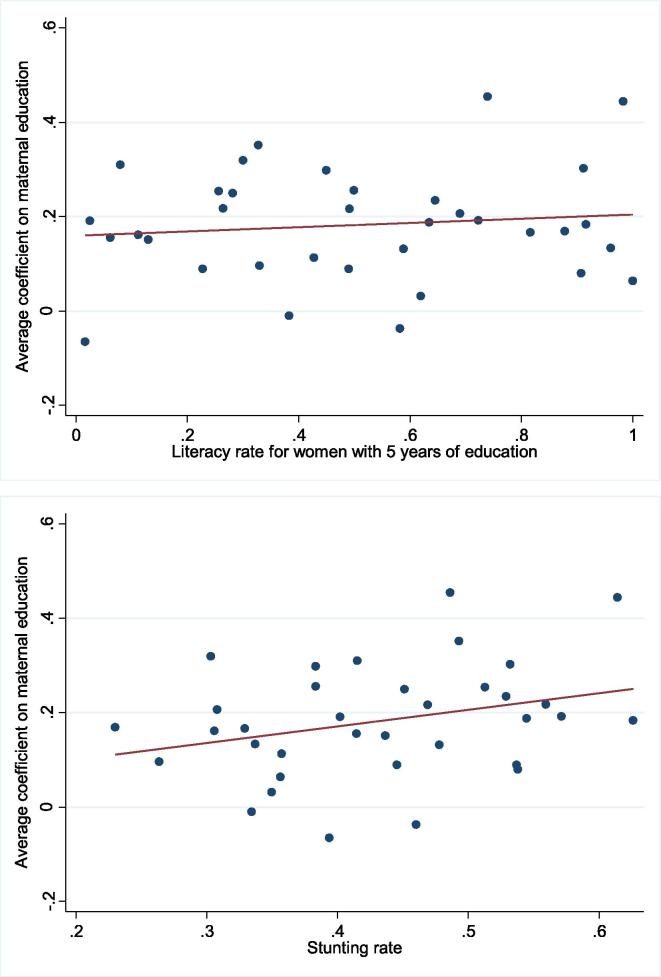
Country-level estimates of the average marginal impacts of maternal education on HAZ^1^ against stunting rates (Panel A) and grade 5 literacy rates (Panel B). *Notes:* Average coefficients refer to the average of coefficients for the 7–9, 10–12 and 13 + education brackets for maternal education.

In Table 5 we present results by some important subsamples. We first split the sample into low- and high-undernutrition burden countries, with the division set at stunting levels of 30% (HAZ < −2). There are many reasons why education might matter less in countries with low stunting. Superficially, there is less variation in HAZ in low-burden countries, and often less variation in education, particularly in the ECA sub-sample. Theoretically, the better provision of public services and higher incomes in low-burden countries might also limit the adverse effects of low education. Consistent with these conjectures, the coefficients on maternal education brackets are much smaller in low-burden countries (Regression 1) than in high-burden countries (Regression 2), typically about one quarter of the size. Figure A1 in the appendix provides further corroboration that the coefficients on maternal education tend to vary positively with national stunting rates.

**Figure 3:**
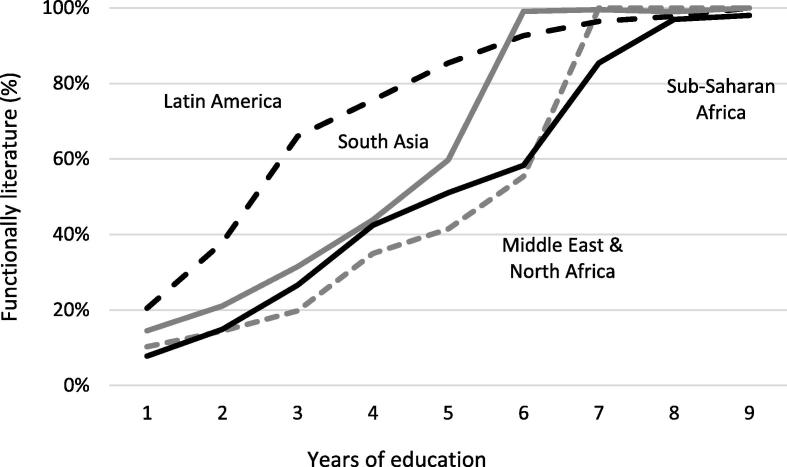
Maternal literacy levels by years of schooling across four developing regions. Notes: Literacy is defined as the ability to read a whole sentence. Regional groups are World Bank classifications: MNA = Middle East and North Africa; LAC = Latin America and Caribbean; SAS = South Asia; SSA = Sub-Saharan Africa. Eastern Europe and Central Asia is excluded because there are very few mothers who have only completed a few years of primary schooling.

The remaining regressions in Table 5 examine the importance of education quality, as proxied by the percentage of mothers with 5 years of education who can read a complete sentence. Figure 3 shows that this measure of quality varies quite markedly across regions. Literacy attainment in Latin America is substantially faster than in the other regions, and notably much better than in the Middle East and North Africa despite the two regions having otherwise similar levels of economic development. In South Asia female literacy attainment is generally better, but unsurprisingly sub-Saharan Africa performs quite poorly (albeit with substantial heterogeneity within the region). Regressions 3 and 4 in Table 5 split the full sample into a lower quality group (Grade 5 literacy < 50%) and a higher quality group (Grade 5 literacy > 50%). The nutritional impacts in the higher quality group are significantly larger than in the lower quality group, though only for maternal education (see also Figure A1 in the appendix). Moreover, the coefficients on 4–6 years and 7–9 years of maternal education are insignificant in the lower quality sample, and substantially smaller in magnitude at higher levels of education. Thus there is some evidence that the quality of education at least matters for maternal education’s impacts; it is possible that the same associations would hold for indicators of paternal education quality, but we do not have the data to test this conjecture.

The regional results in Table 6 show variation even with low- and high-burden regions, but the pattern of results seems consistent with educational quality, as proxied by maternal literacy (regional Grade 5 literacy rates are reported at the bottom of Table 6). For example, we do not find any significant marginal effects of parental education on nutrition in the Middle East and North Africa, a low-burden, low-quality region.^14^ In contrast, Latin America is a low-burden but high-quality region, and there are reasonably high returns to maternal education, but not to paternal education. The maternal education coefficients in the sub-Saharan Africa sample—a high-burden, low-quality region - are similar in magnitude to the Latin America sample, while the paternal education coefficients are mostly significant but still small in magnitude. South Asia is a high-burden, high-quality region, and the coefficients on maternal education are substantially larger than in other regions. For example, children of mothers with tertiary education are predicted to have HAZ scores 0.47 standard deviations larger than children of mothers with little or no education (all else equal), whereas the analogous coefficients for sub-Saharan Africa and Latin America are 0.25 standard deviations. Moreover, even paternal education yields reasonably large coefficients in South Asia, of an order similar in magnitude to the maternal education coefficients in sub-Saharan Africa and Latin America.

We also estimated regressions for each country in the sample, based on pooling multiple DHS rounds within a country (Table 7). We did this partly to gauge variation in coefficients sizes and partly to gauge the extent of heterogeneity among country-specific analyses. Table 6 reports a summary of these results. The estimated returns to education vary appreciably in the country-specific regressions. Even after excluding Eastern European and Central Asian countries (which consistently yield large negative outliers in terms of the education coefficients) and countries with fewer than 2,000 observations, the coefficients for higher education for mothers are significant in less than half of the 40 remaining countries. The same is true for paternal education coefficients. However, the average magnitude of the coefficients is larger for maternal education than it is for paternal education, consistent with results from the pooled sample.

## 5 Discussion

The results in this paper inform the longstanding question of whether parental education substantially affects the health of the next generation, and whether maternal education has larger effects than paternal education. Unlike previous research on the nutritional impacts of parental education, we provide more rigorous and extensive tests based on proxy controls for cluster, household and individual unobservables that might bias conventional least squares specifications. Like other papers (Vollmer et al., 2016, Desai and Alva, 1998) we find that the addition of cluster fixed effects substantially reduces the estimated returns to parental education, possibly reflecting a correlation of education and health infrastructure. Adding educational ranks further reduces parental education coefficients, with the direction of attenuation consistent with Duflo’s (2012) hypothesis that least squares maternal education coefficients are likely to be biased in discriminatory environments. Even so, the additional attenuation from adding educational ranks is modest, and the results still suggest that maternal education has a significantly larger impact on nutrition than paternal education, and that maternal education is characterized by increasing returns, with the estimated impacts of 10–12 years and 13-plus years being disproportionately larger than those of primary or middle school.

What might explain these results? One explanation of the small coefficient of primary schooling (except insofar as primary education is a prerequisite for secondary education) is the apparent low quality of primary education services and, thus, a generally poor translation of primary school attendance into nutritionally relevant learning outcomes. Figure 3 demonstrates the strikingly poor literacy attainment for mothers who report completely several years of primary education, particularly in MENA and sub-Saharan Africa.

Of course, these literacy data only really tell us about schooling quality in primary school. The fact that we find evidence of increasing returns to women’s education—and large differences between maternal and paternal education coefficients at higher levels—suggests that health care messages and behavioral changes are complex enough to require more than just the most basic literacy and numeracy skills. Keeping girls in school longer can delay age of marriage and first birth, reduce the demand for children, and empower women to make decisions that they might not otherwise make, such as having few and more evenly spaced births, and making better use of modern health services.

To explore this hypothesis further we used regressions similar to regressions 3 and 4 in Table 3 to test whether parental education is associated with a range of behaviors that can influence nutrition (that is, we continue to control for cluster fixed effects and education ranks). Consistent with the results reported in Table 3, we find that maternal education often has much stronger associations with these outcomes than paternal education (Appendix Table 11, Table 12).^15^ Specifically, maternal education has significantly stronger associations with the number of children ever born (i.e. fertility), children’s dietary diversity (but not maternal dietary diversity), antenatal and post-natal care (columns 1 and 2), and a woman’s ability to participate in decisions about her own health care (by herself or jointly). Interestingly, we find no advantage of maternal education in affecting decisions to vaccinate her children or exclusive breastfeeding in the first few months of life, both of which may be more associated with child mortality than child growth outcomes. Indeed, this finding may partly explain Fafchamps and Shilpi’s (2015) conclusion that maternal education is not a stronger predictor of child mortality outcomes than paternal education. One might speculate that, at any given education level, fathers care just as much about keeping their children alive as mothers do, but devote less attention to more mundane day-to-day child care practices like feeding children an appropriately diverse diet. In sum, the exploratory results reported in Table 11, Table 12 point to a stronger role for women’s education in influencing a range of nutrition-relevant behaviors.

Finally, we broach the policy relevant question of how much further expansion of education could contribute to improved nutrition in high-burden countries. Table 8 uses separate stunting regression models for South Asia and sub-Saharan Africa to estimate the predicted reductions in stunting from three scenarios: achieving universal completion of primary school (6 years), middle school (9 years), and upper secondary (12 years). We recognize that the scenarios are only illustrative given that the results used here are derived from cross sectional regression estimates that are not strictly causal, meaning that the true coefficients could be smaller (because of additional omitted variables) or larger (because our approach may over-control for location effects and potentially exogenous effects captured by cohort ranks). Moreover, achieving universal upper secondary education in these regions would be very expensive and unrealistic in the near future. In Africa alone, universal primary school completion is expected to cost USD 18 billion, while universal middle school completion will cost USD 24 billion (UNESCO, 2012).

With these caveats in mind, these simulations suggest that achieving universal primary schooling—now a widely adopted policy in Africa and Asia—would only reduce stunting modestly, by just 2.5 percentage points. Mandatory middle schooling has a larger estimated impact, reducing stunting rates by 6 percentage points, while the much more ambitious target of universal upper secondary completion would reduce stunting by 10.3 percentage points. Gender differences in the coefficients and in baseline schooling attainment mean that most of these gains come from expanding girls’ education.

The simulations therefore suggest that the expected nutritional gains from schooling investments are moderately large, but certainly much smaller than more naïve regression models would suggest. Of course, in both these high-burden regions it might be eminently feasible to increase the nutritional returns to any given level of schooling through improvements in the generic quality of schooling, as well as through improvements in the health and nutritional content of the education curriculum (Glewwe, 1999). But while substantial research has examined the impacts of nutritional education programs targeted at mothers (Imdad et al., 2011, Lassi et al., 2013), scarcely any research has examined the benefits of targeting nutritional information to adolescent girls—future mothers—through formal schooling (Glewwe, 1999). More research on this issue is sorely needed.

## Conflict of interest statement

We, the authors, declare that we have no conflicts of interest that may compromise the objectivity and rigor of this research.

## Figures and Tables

**Table 9:** List of countries and rounds from the various DHS surveys

Country	Observations per country	Survey years	Observations per survey
Albania	916	2009	916
Armenia	1,624	2000	927
		2005	697
Azerbaijan	1,053	2006	1053
Bangladesh	16,424	1997	2551
		2000	2869
		2004	3414
		2007	3046
		2011	4544
Burkina Faso	7,932	2003	4332
		2010	3600
Benin	7,731	1996	571
		2001	2103
		2012	5057
		2012	5057
Bolivia	12,692	1998	3403
		2003	5144
		2008	4145
Brazil	2,086	1996	2086
Burundi	1,801	2010	1801
Congo, Dem. Rep.	5,714	2007	1606
		2014	4108
Central African Republic	533	1995	533
Chad	2,360	2004	2360
Congo, Republic	3,771	2005	1718
		2012	2053
Cote d'Ivoire	1,427	2012	1427
Cameroon	3,875	2004	1463
		2011	2412
Colombia	19,052	1995	2351
		2000	2184
		2005	6245
		2010	8272
Dominican Republic	1,609	2013	1609
Egypt	28,180	1995	5966
		2000	5957
		2003	3560
		2005	7311
		2008	5386
Ethiopia	12,435	2000	4861
		2005	2172
		2011	5402
Ghana	4,153	1998	1394
		2003	1542
		2008	1217
Guinea	5,251	1999	2291
		2005	1348
		2012	1612
Guatemala	6,526	1995	4361
		1999	2165
Guyana	641	2009	641
Haiti	5,974	2000	2691
		2006	1296
		2012	1987
India	24,127	2006	24,127
Jordan	9,866	1997	3294
		2002	2803
		2012	3769
Kenya	7,339	1998	2262
		2003	2318
		2009	2759
		2009	2759
Cambodia	6,096	2000	2034
		2005	1963
		2010	2099
Kazakhstan	545	1995	210
		1999	335
Comoros	1,494	1996	219
		2012	1275
Kyrgyz Republic	2,459	1997	292
		2012	2167
Liberia	3,543	2007	2064
		2013	1479
Lesotho	1,394	2004	642
		2009	752
Morocco	3,029	2004	3029
Moldova	721	2005	721
Madagascar	5,462	1997	641
		2004	2169
		2009	2652
		2009	2652
Mali	8,166	2006	5572
		2013	2594
Malawi	11,157	2000	4626
		2004	4013
		2010	2518
Mozambique	9,209	1997	703
		2003	3826
		2011	4680
Nicaragua	7,068	1998	3856
		2001	3212
Nigeria	25,856	1999	379
		2003	2211
		2008	10,258
		2013	13,008
Niger	4,478	2006	1879
		2012	2599
Nepal	8,876	1996	959
		2001	3457
		2006	3069
		2011	1391
Peru	34,893	1996	8109
		2000	6387
		2008	5662
		2009	5079
		2010	4790
		2011	4866
Pakistan	1,859	2013	1859
Rwanda	7,183	2000	3063
		2005	1856
		2010	2264
Sierra Leone	3,114	2008	974
		2013	2140
Senegal	1,825	2011	1825
Sao Tome and Principe	878	2009	878
Tajikistan	2,548	2012	2548
Timor-Leste	4,578	2010	4578
Tanzania	7,202	2005	3656
		2010	3546
Uganda	4,668	2001	2462
		2006	1159
		2011	1047
Uzbekistan	297	1996	297
Zambia	7,881	1996	2657
		2002	2631
		2007	2593
Zimbabwe	5,421	1999	1332
		2006	2071
		2011	2018

**Table 10:** Regressions of nutrition indicators against parental education for the full sample of children 0–59 months

Regression number	1	2	3	4
Dependent variable	HAZ	HAZ	HAZ	Stunting
Country-varying controls?	Yes	Yes	Yes	Yes
Fixed effects	No	Yes	Yes	Yes
Education rank included?	No	No	Yes	Yes
*Maternal education*				
4–6 years	0.117^∗∗∗^	0.043^∗∗∗^	0.023^∗∗∗^	−0.008^∗∗∗^
	(0.007)	(0.006)	(0.008)	(0.002)
7–9 years	0.190^∗∗∗^	0.076^∗∗∗^	0.045^∗∗∗^	−0.020^∗∗∗^
	(0.007)	(0.007)	(0.010)	(0.003)
10–12 years	0.322^∗∗∗^	0.163^∗∗∗^	0.120^∗∗∗^	−0.039^∗∗∗^
	(0.008)	(0.008)	(0.013)	(0.004)
13 plus	0.460^∗∗∗^	0.250^∗∗∗^	0.190^∗∗∗^	−0.048^∗∗∗^
	(0.011)	(0.011)	(0.017)	(0.005)

*Paternal*				
4–6 years	0.065^∗∗∗^	0.036^∗∗∗^	0.010	−0.007^∗∗∗^
	(0.007)	(0.007)	(0.008)	(0.002)
7–9 years	0.110^∗∗∗^	0.074^∗∗∗^	0.036^∗∗∗^	−0.015^∗∗∗^
	(0.007)	(0.007)	(0.009)	(0.003)
10–12 years	0.168^∗∗∗^	0.118^∗∗∗^	0.062^∗∗∗^	−0.023^∗∗∗^
	(0.007)	(0.007)	(0.012)	(0.004)
13+ years	0.239^∗∗∗^	0.164^∗∗∗^	0.094^∗∗∗^	−0.025^∗∗∗^
	(0.009)	(0.009)	(0.015)	(0.004)

*Cohort-region educational ranks*				
Mother's rank			0.069^∗∗∗^	−0.022^∗∗∗^
			(0.015)	(0.005)
Father's rank			0.089^∗∗∗^	−0.023^∗∗∗^
			(0.015)	(0.005)

*Tests*				
Larger maternal education effects?^a^	Yes (all brackets)	Yes (10–12; 13+)	Yes (10–12; 13+)	Yes (10–12; 13+)
Significant non-linearity?^b^	Yes (women); No (men)	Yes	Yes	
*N*	668,066	668,066	668,066	668,066

*Notes:* Cluster-robust standard errors reported in parenthesis. ∗, ∗∗, and ∗∗∗ indicate significance at the 10%, 5% and 1% significance levels respectively. See text for a description of the models and variables. a. This test is a Wald test comparing maternal and paternal coefficients across categories. b. This is a test of coefficient proportionality.

**Table 11:** Regressions of child growth indicators against parental education under alternative specifications, after excluding household wealth

Regression number	1	2	3	4
Dependent variable	HAZ	HAZ	HAZ	Stunting
Country-varying controls?	Yes	Yes	Yes	Yes
Community Fixed effects	No	Yes	Yes	Yes
Education rank included?	No	No	Yes	Yes

*Maternal education*				
4–6 years	0.160^∗∗∗^	0.068^∗∗∗^	0.039^∗∗∗^	−0.014^∗∗∗^
	0.008	0.008	0.009	0.003
7–9 years	0.287^∗∗∗^	0.122^∗∗∗^	0.079^∗∗∗^	−0.032^∗∗∗^
	0.008	0.008	0.011	0.004
10–12 years	0.488^∗∗∗^	0.232^∗∗∗^	0.172^∗∗∗^	−0.060^∗∗∗^
	0.01	0.009	0.014	0.005
13 plus	0.684^∗∗∗^	0.326^∗∗∗^	0.246^∗∗∗^	−0.066^∗∗∗^
	0.013	0.013	0.02	0.007

*Paternal*				
4–6 years	0.087^∗∗∗^	0.053^∗∗∗^	0.028^∗∗∗^	−0.012^∗∗∗^
	0.008	0.008	0.009	0.003
7–9 years	0.165^∗∗∗^	0.104^∗∗∗^	0.066^∗∗∗^	−0.025^∗∗∗^
	0.008	0.008	0.011	0.004
10–12 years	0.255^∗∗∗^	0.167^∗∗∗^	0.111^∗∗∗^	−0.039^∗∗∗^
	0.009	0.008	0.014	0.005
13+ years	0.349^∗∗∗^	0.238^∗∗∗^	0.165^∗∗∗^	−0.049^∗∗∗^
	0.011	0.011	0.019	0.006

*Cohort-region educational ranks*				
Mother's rank			0.097^∗∗∗^	−0.029^∗∗∗^
			0.018	0.006
Father's rank			0.088^∗∗∗^	−0.026^∗∗∗^
			0.019	0.006

*Tests*				
Larger maternal education effects?^a^	Yes (all brackets)	Yes (10–12; 13+)	Yes (10–12; 13+)	Yes (10–12; 13+)
Significant non-linearity?^b^	Yes: Women	Yes	Yes	Yes: Women
N	376,992	376,992	376,992	376,992

Notes: Cluster-robust standard errors reported in parenthesis. Clustering is at the enumeration level ∗, ∗∗, and ∗∗∗ indicate significance at the 10%, 5% and 1% significance levels respectively. See text for a description of the models and variables. a. This test is a Wald test comparing maternal and paternal coefficients across categories. b. This is a test of coefficient proportionality.

**Table 12:** Exploring potential explanations: fertility and dietary indicators

Dependent variable (unit)	Number of children (number)	Child dietary diversity score (0–7)	Maternal diet diversity (0–7)	Exclusive breastfeeding months 0–5 (0/1)	Mother has low BMI (0/1)
Larger maternal benefits?^a^	Yes	Yes	No	No	No

*Maternal education*					
4–6 years	−0.261^∗∗∗^	0.078^∗∗∗^	0.029	0.000	−0.006^∗∗∗^
	(0.010)	(0.026)	(0.024)	(0.005)	(0.002)
7–9 years	−0.446^∗∗∗^	0.129^∗∗∗^	0.045	−0.004	−0.006^∗∗^
	(0.013)	(0.035)	(0.030)	(0.006)	(0.002)
10–12 years	−0.830^∗∗∗^	0.151^∗∗∗^	0.065	−0.006	−0.005^∗^
	(0.016)	(0.047)	(0.040)	(0.008)	(0.003)
13+ years	−1.393^∗∗∗^	0.185^∗∗∗^	0.106^∗∗^	−0.012	−0.009^∗∗^
	(0.022)	(0.061)	(0.052)	(0.010)	(0.004)

*Paternal education*					
4–6 years	−0.122^∗∗∗^	0.023	0.032	0.002	−0.009^∗∗∗^
	(0.010)	(0.026)	(0.023)	(0.005)	(0.002)
7–9 years	−0.166^∗∗∗^	0.038	0.069^∗∗^	0.005	−0.014^∗∗∗^
	(0.012)	(0.031)	(0.028)	(0.005)	(0.002)
10–12 years	−0.277^∗∗∗^	0.051	0.080^∗∗^	0.004	−0.016^∗∗∗^
	(0.016)	(0.041)	(0.035)	(0.007)	(0.003)
13 + years	−0.352^∗∗∗^	0.044	0.129^∗∗∗^	0.005	−0.021^∗∗∗^
	(0.020)	(0.052)	(0.045)	(0.008)	(0.004)

*Education ranks*					
Maternal	0.058^∗∗∗^	0.127^∗∗^	0.145^∗∗∗^	0.015	−0.007^∗^
	(0.021)	(0.058)	(0.048)	(0.009)	(0.004)
Paternal	0.055^∗∗∗^	0.096^∗^	0.077^∗^	−0.007	−0.005
	(0.020)	(0.053)	(0.046)	(0.009)	(0.004)
					
R-squared	0.557	0.29	0.019	0.061	0.014
N	668,066	103,936	69,432	60,109	603,624

*Source:* Authors’ estimates from DHS data. See text for details.

*Notes:* Cluster-robust standard errors reported in parenthesis. ∗, ∗∗, and ∗∗∗ indicate significance at the 10%, 5% and 1% significance levels respectively. The regression models are analogous to regression 3 from Table 3.

**Table 13:** Exploring potential explanations: maternal and child health indicators

Dependent variable (unit)	Received antenatal care (0/1)	Received postnatal care (0/1)	Proportion of 9 vaccinations (0–1)	Mother decides child health (0/1)	Mother decides own health (0/1)
Larger maternal effects?^a^	Yes	Yes (10–12; 13+)	No	No	Yes

*Maternal education*					
4–6 years	0.033^∗∗∗^	0.033^∗∗∗^	0.014^∗∗∗^	0.009^∗∗∗^	0.014^∗∗∗^
	(0.003)	(0.003)	(0.002)	(0.003)	(0.003)
7–9 years	0.054^∗∗∗^	0.054^∗∗∗^	0.018^∗∗∗^	0.010^∗∗∗^	0.022^∗∗∗^
	(0.003)	(0.003)	(0.002)	(0.004)	(0.004)
10–12 years	0.071^∗∗∗^	0.071^∗∗∗^	0.024^∗∗∗^	0.004	0.053^∗∗∗^
	(0.004)	(0.004)	(0.003)	(0.005)	(0.005)
13+ years	0.092^∗∗∗^	0.092^∗∗∗^	0.023^∗∗∗^	−0.001	0.083^∗∗∗^
	(0.006)	(0.006)	(0.003)	(0.006)	(0.007)

*Paternal education*					
4–6 years	0.029^∗∗∗^	0.029^∗∗∗^	0.017^∗∗∗^	0.010^∗∗∗^	0.000
	(0.003)	(0.003)	(0.002)	(0.003)	(0.003)
7–9 years	0.044^∗∗∗^	0.044^∗∗∗^	0.021^∗∗∗^	0.014^∗∗∗^	0.005
	(0.003)	(0.003)	(0.002)	(0.004)	(0.004)
10–12 years	0.049^∗∗∗^	0.049^∗∗∗^	0.026^∗∗∗^	0.020^∗∗∗^	0.010^∗∗^
	(0.004)	(0.004)	(0.002)	(0.005)	(0.005)
13+ years	0.064^∗∗∗^	0.064^∗∗∗^	0.032^∗∗∗^	0.023^∗∗∗^	0.012^∗∗^
	(0.005)	(0.005)	(0.003)	(0.006)	(0.006)

*Education ranks*					
Maternal	0.086^∗∗∗^	0.086^∗∗∗^	0.047^∗∗∗^	0.030^∗∗∗^	0.024^∗∗∗^
	(0.005)	(0.005)	(0.003)	(0.007)	(0.006)
Paternal	0.032^∗∗∗^	0.032^∗∗∗^	0.017^∗∗∗^	−0.010	−0.003
	(0.005)	(0.005)	(0.003)	(0.006)	(0.006)

*R*-Squared	0.034	0.034	0.289	0.007	0.011
N	508,513	508,513	528,278	201,800	516,298

*Source:* Authors’ estimates from DHS data. See text for details.

*Notes:* Cluster-robust standard errors reported in parenthesis. ∗, ∗∗, and ∗∗∗ indicate significance at the 10%, 5% and 1% significance levels respectively. The regression models are analogous to regression 3 from Table 3. a. This test is a Wald test comparing maternal and paternal coefficients across categories.

**Table 1:** Sample descriptive statistics

Variable	Observations	Mean	Std. Dev.	Min	Max
HAZ score	376,992	−1.64	1.47	−5.00	5.00
Moderate stunting, HAZ < −2	376,992	0.40	0.49	0.00	1.00
Women's education (years)	376,992	4.86	4.77	0.00	25.00
Paternal education (years)	376,992	6.13	5.06	0.00	32.00
Wealth index (0–10)	376,992	4.04	3.05	0.30	9.03
Female child (0/1)	376,992	0.49	0.50	0.00	1.00
Rural residence (0/1)	376,992	0.65	0.48	0.00	1.00
Child age (months)	376,992	41.64	10.09	25.00	59.00
Mother's height (cm)	376,992	155.79	7.12	100.00	216.00
Mother's age (years)	376,992	29.84	6.67	15.00	49.00
Father's age (years)	376,992	36.62	9.07	10.00	95.00

*Source*: Authors’ estimates from DHS data. See text for details.

**Table 2:** HAZ scores and parental education (means) by developing regions and high/low-burden country groupings

Region (number of countries, N)	Sample size (% total)	HAZ score	Stunting Rate (%)	Maternal education (years)	Paternal education (years)
*Regions*					
East Asia (*N* = 2)	10,674 (2.8%)	−2.10	55.3%	4.29	5.39
Eastern Europe & Central Asia (*N* = 8)	10,163 (2.7%)	−1.06	23.1%	10.69	11.21
Latin America & Caribbean (*N* = 8)	84,015 (22.3%)	−1.33	28.6%	6.73	7.62
Middle East & North Africa (M = 3)	41,075 (10.9%)	−1.07	22.4%	6.70	8.01
South Asia (*N* = 4)	51,286 (13.6%)	−1.99	51.6%	4.15	5.79
Sub-Saharan Africa (*N* = 32)	179,779 (47.7%)	−1.83	47.0%	3.47	4.85

*Undernutrition burdens*					
Low burden: Stunting < 25% (*N* = 15)	74,154 (19.7%)	−1.02	0.20	7.38	8.36
High burden: Stunting < 25% (*N* = 41)	302,838 (80.3%)	−1.80	0.45	4.24	5.58
Total (56)	376,992 (100%)	−1.64	0.40	4.86	6.13

*Source:* Authors’ estimates from DHS data. See text for details. Note: East Asia includes only relatively 2 small countries, Cambodia and Timor–Leste.

**Table 3:** Regressions of child growth indicators against parental education under alternative specifications

Regression number	1	2	3	4
Dependent variable	HAZ	HAZ	HAZ	Stunting
Country-varying controls?	Yes	Yes	Yes	Yes
Community Fixed effects	No	Yes	Yes	Yes
Education rank included?	No	No	Yes	Yes

*Maternal education*				
4–6 years	0.122^∗∗∗^	0.048^∗∗∗^	0.027^∗∗∗^	−0.010^∗∗∗^
	(0.008)	(0.008)	(0.009)	(0.003)
7–9 years	0.208^∗∗∗^	0.087^∗∗∗^	0.055^∗∗∗^	−0.024^∗∗∗^
	(0.008)	(0.008)	(0.011)	(0.004)
10–12 years	0.361^∗∗∗^	0.182^∗∗∗^	0.138^∗∗∗^	−0.048^∗∗∗^
	(0.009)	(0.009)	(0.014)	(0.005)
13 plus	0.522^∗∗∗^	0.268^∗∗∗^	0.209^∗∗∗^	−0.055^∗∗∗^
	(0.013)	(0.013)	(0.02)	(0.007)

*Paternal*				
4–6 years	0.057^∗∗∗^	0.033^∗∗∗^	0.012	−0.007^∗∗^
	(0.008)	(0.008)	(0.009)	(0.003)
7–9 years	0.110^∗∗∗^	0.077^∗∗∗^	0.046^∗∗∗^	−0.019^∗∗∗^
	(0.008)	(0.008)	(0.011)	(0.004)
10–12 years	0.174^∗∗∗^	0.128^∗∗∗^	0.081^∗∗∗^	−0.029^∗∗∗^
	(0.009)	(0.008)	(0.014)	(0.005)
13+ years	0.247^∗∗∗^	0.184^∗∗∗^	0.124^∗∗∗^	−0.036^∗∗∗^
	(0.011)	(0.011)	(0.019)	(0.006)

*Cohort-region educational ranks*				
Mother's rank			0.072^∗∗∗^	−0.021^∗∗∗^
			(0.018)	(0.006)
Father's rank			0.073^∗∗∗^	−0.020^∗∗∗^
			(0.019)	(0.006)

*Tests*				
Larger maternal education effects?^a^	Yes (all brackets)	Yes (10–12; 13+)	Yes (10–12; 13+)	Yes (10–12; 13+)
Significant non-linearity?^b^	Yes: Women	Yes	Yes	Yes: Women
*N*	376,992	376,992	376,992	376,992

*Notes:* Cluster-robust standard errors reported in parenthesis. Clustering is at the enumeration level ∗, ∗∗, and ∗∗∗ indicate significance at the 10%, 5% and 1% significance levels respectively. See text for a description of the models and variables. a. This test is a Wald test comparing maternal and paternal coefficients across categories; significance refers to the 10% level or lower. b. This is a test of coefficient proportionality; significance refers to the 10% level or lower.

**Table 4:** Testing for significant interactions between parental education and socioeconomic status (asset-based poverty)

Regression number	1	2
Dependent variable	HAZ	Stunting
Country-varying controls?	Yes	Yes
Community Fixed effects	No	Yes
Education rank included?	No	No
Mother's education (years)	0.019^∗∗∗^	−0.006^∗∗∗^
	(0.002)	(0.001)
Partner's education (years)	0.011^∗∗∗^	−0.003^∗∗∗^
	(0.002)	(0.001)
Asset poor = 1	−0.135^∗∗∗^	0.047^∗∗∗^
	(0.011)	(0.004)
Mother's education * Asset poor	−0.009^∗∗∗^	0.002^∗∗∗^
	(0.002)	(0.001)
Father's education * Asset poor	0.001	0.001
	(0.001)	(0.001)
Difference between genders significant at 10% level if asset poor?	No	No
*N*	376,992	376,992

*Notes:* Cluster-robust standard errors reported in parenthesis. Clustering is at the enumeration level ∗, ∗∗, and ∗∗∗ indicate significance at the 10%, 5% and 1% significance levels respectively. The “Asset poverty” indicator is a dichotomous variable equal to one if a household’s wealth score is below the 40th percentile. The wealth score is constructed from the first principal component of a common set of asset indicators: electricity access, radio ownership, TV ownership, improved floor material, ownership of a flush toilet and ownership of a basic toilet. See the text for a description of the models and other variables used in these regressions.

**Table 5:** Testing heterogeneity of HAZ results across low and high undernutrition burden samples, and low and high education quality samples

Sample	Low stunting burden: stunting < 25%	High stunting burden: stunting > 25%	Low education quality: Gr 5 literacy < 50%	High education quality: Gr 5 literacy > 50%
Dependent variable	HAZ	HAZ	HAZ	HAZ
Country-varying controls?	Yes	Yes	Yes	Yes
Community Fixed effects	Yes	Yes	Yes	Yes
Education rank included?	Yes	Yes	Yes	Yes
*Maternal education*				
4–6 years	0.020	0.028^∗∗∗^	−0.004	0.048^∗∗∗^
	(0.020)	(0.010)	(0.017)	(0.011)
7–9 years	0.002	0.063^∗∗∗^	0.031	0.074^∗∗∗^
	(0.024)	(0.013)	(0.020)	(0.014)
10–12 years	0.055^∗^	0.165^∗∗∗^	0.099^∗∗∗^	0.173^∗∗∗^
	(0.031)	(0.017)	(0.026)	(0.018)
13 plus	0.074^∗^	0.277^∗∗∗^	0.163^∗∗∗^	0.246^∗∗∗^
	(0.043)	(0.022)	(0.035)	(0.023)

*Paternal education*				
4–6 years	−0.010	0.017^∗^	0.022	0.006
	(0.019)	(0.010)	(0.016)	(0.011)
7–9 years	0.004	0.056^∗∗∗^	0.041^∗∗^	0.044^∗∗∗^
	(0.024)	(0.012)	(0.019)	(0.013)
10–12 years	−0.012	0.107^∗∗∗^	0.061^∗∗^	0.093^∗∗∗^
	(0.030)	(0.016)	(0.024)	(0.018)
13+ years	−0.018	0.171^∗∗∗^	0.137^∗∗∗^	0.122^∗∗∗^
	(0.040)	(0.021)	(0.032)	(0.023)

Significant gender differences?^a^	No	Yes: 10–12; 13+	No:	Yes: 4–6; 10–12; 13+
Significant non-linearity?^b^	Yes (women)	Yes (women)	Yes: Both sexes	Yes: Both sexes
Sample size	74,154	302,838	158,138	208,691

Notes: Cluster-robust standard errors reported in parenthesis. Clustering is at the enumeration level. ∗, ∗∗, and ∗∗∗ indicate significance at the 10%, 5% and 1% significance levels respectively. The regression models are analogous to regression 3 from Table 3. a. This test is a Wald test comparing maternal and paternal coefficients across categories; significance refers to the 10% level or lower. b. This is a test of coefficient proportionality; significance refers to the 10% level or lower.

**Table 6:** Testing heterogeneity of HAZ results across major regions

Sample	Middle East & North Africa	Latin America & Caribbean	South Asia	Sub-Saharan Africa
Dependent variable	HAZ	HAZ	HAZ	HAZ
Country-varying controls?	Yes	Yes	Yes	Yes
Community Fixed effects	Yes	Yes	Yes	Yes
Education rank included?	Yes	Yes	Yes	Yes

*Maternal education*				
4–6 years	0.038	0.050^∗∗∗^	0.055^∗∗^	0.012
	(0.030)	(0.015)	(0.025)	(0.015)
7–9 years	−0.006	0.099^∗∗∗^	0.092^∗∗∗^	0.041^∗∗^
	(0.032)	(0.021)	(0.030)	(0.018)
10–12 years	0.064	0.163^∗∗∗^	0.205^∗∗∗^	0.133^∗∗∗^
	(0.040)	(0.028)	(0.041)	(0.024)
13 plus	0.088	0.255^∗∗∗^	0.375^∗∗∗^	0.241^∗∗∗^
	(0.056)	(0.036)	(0.055)	(0.037)

*Paternal education*				
4–6 years	−0.008	−0.003	0.019	0.015
	(0.028)	(0.015)	(0.025)	(0.014)
7–9 years	0.002	0.022	0.078^∗∗∗^	0.046^∗∗∗^
	(0.033)	(0.020)	(0.029)	(0.017)
10–12 years	−0.019	0.045^∗^	0.151^∗∗∗^	0.083^∗∗∗^
	(0.044)	(0.027)	(0.039)	(0.022)
13+ years	0.004	0.031	0.230^∗∗∗^	0.166^∗∗∗^
	(0.060)	(0.035)	(0.051)	(0.029)

	63%	90%	99%	53%
Significant gender differences?^a^	No	Yes: All levels	Yes: 10–12	Yes: 10–12
Significant non-linearity?^b^	Yes (women)	No (marginal)	Yes: Both sexes	Yes: Both sexes
Sample size	41,075	84,015	51,286	179,779

*Notes:* Cluster-robust standard errors reported in parenthesis. Clustering is at the enumeration level. ∗, ∗∗, and ∗∗∗ indicate significance at the 10%, 5% and 1% significance levels respectively. The regression models are analogous to regression 3 from Table 3. a. This test is a Wald test comparing maternal and paternal coefficients across categories; significance refers to the 10% level or lower. b. This is a test of coefficient proportionality; significance refers to the 10% level or lower.

**Table 7:** Summary of country-specific HAZ score regression results for 40 countries^a^

	Education bracket	Countries with significant coefficients at 10% level	Average coefficient	Standard deviation of Coefficients
Mothers	4–6 years	9	0.05	0.14
	7–9 years	7	0.08	0.17
	10–12 years	18	0.17	0.23
	13+ years	16	0.26	0.32

Fathers	4–6 years	4	0.01	0.29
	7–9 years	5	0.04	0.12
	10–12 years	15	0.07	0.15
	13+ years	15	0.14	0.18

*Notes:* The regressions used to make this table are similar in structure to regression 3 of Table 3, but are run for each country, including multiple DHS rounds within a country when available. a. Eastern European and Central Asian countries are excluded from these calculations, since they are large negative outliers. We also exclude countries with fewer than 2,000 observations.

**Table 8:** Estimating the reduction in stunting from increasing various education policies in high-burden countries in South Asia and sub-Saharan Africa

	Alternative scenarios		
	Universal primary school completion (6 years)	Universal middle school completion (9 years)	Universal *upper secondary* completion (12 years)
Reduction from both sexes	−2.46%	−6.04%	−10.32%
Reduction from girls' education	−1.45%	−3.90%	−6.23%
Reduction from boys education	−1.01%	−2.15%	−4.10%

*Source:* Authors’ estimates from DHS data. See text for details.

*Notes:* These estimates are based on three steps. First, we separately estimated regressions of stunting against education levels for high-undernutrition burden countries in South Asia and sub-Saharan Africa, analogous to the HAZ regressions in Table 5. Second, we estimate mean levels of education by bracket for South Asian and sub-Saharan African DHS surveys post-2005 and parents aged 20–29 years of age (i.e. the most recent generation of parents). Third, we estimate counterfactual distributions of education for the current generation: universal primary parents with 0–6 years of education are now assigned 7–9 years; universal secondary, where parents with 0–9 years of education are now assigned 10–12 years of education; universal tertiary education, where the whole population is assigned 13-plus years of education. Fourth, we calculate predicted changes in stunting rates from these counterfactual distributions and the estimated coefficients derived from step 1. In each scenario we always hold the proportion of parents from higher education levels constant (e.g. in scenario 1 we reallocate parents in the 0–3 and 4–6 year brackets to the 7–9 year bracket, but keep the 10–12 and 13 + proportions the same). Note, also, that since the number of children 0–14 in South Asia and sub-Saharan Africa is relatively similar, we equally weight the results from each region to derive an aggregate for both regions.

## Appendix A.

Table 9, Table 10, Table 11, Table 12, Table 13 and Figure A1.

## Footnotes

1 On a more pragmatic level, randomizing education interventions would often be politically unacceptable because of legitimate equity concerns.

2 Throughout the text we use the terms maternal and paternal, even though the data include a mix of biological and non-biological parents.

3 See also Ferre (2009), who provides quasi-experimental of reduced fertility rates from a 1985 education reform.

4 A quote from form World Bank Chief Economist Larry Summers (1994) is indicative of this widely held view: “The evidence that mothers channel much more of their income to expenditures on children than their husbands do is overwhelming. Education also increases the willingness to seek medical care and improves sanitation practices. Small wonder that the children of more educated women are much more likely to grow up healthy.” In contrast, Duflo (2012) writes: “many believe that a special effort is needed to educate girls … Unfortunately, the evidence for this is not as strong as is commonly believed … the automatic presumption that female education is more important than male education for child mortality and for other children outcomes may need to be revised: it seems that both matter.”

5 In contrast, an analogous study of Taiwan by Chou, Liu, Grossman, and Joyce (2010) finds the coefficient of father’s education to be only 59% of that for mothers in estimates of neo-natal mortality and 87% for estimates of birth weight.

6 Our sampling strategy involved including as many surveys and countries as possible. We dropped surveys and observations that lacked information on key variables, however, particularly anthropometrics, parental education and age, maternal height, and the asset indicators required to construct a wealth index.

7 This sample size was arrived at after dropping approximately 36,000 observations for which there were no data on paternal education. Since this amounted to less than 5% of the total sample, we dropped these observations from our sample. The inclusion of these observations made no substantive quantitative or qualitative differences to the coefficients on the women’s education brackets in regressions that did not include parental education.

8 Maternal education questions are answered by mothers, while paternal education questions are sometimes answered by fathers themselves (if they are interviewed), but sometimes answered by mothers.

9 This not only addresses program placement it also controls for community education levels. These have been shown to have an independent impact on nutrition over the impact of an individual’s education (Moestue & Huttly, 2008). This community effect is, however, still a reflection of education policy.

10 While Fafchanps and Shilpi focus on an upward bias in the coefficient of education due to endogenous choice by indiviudals or households they recognize that the approach may over compensate if the ranking partially reflects temporal or spatial heterogeneity that is casual but exogenous to the household.

11 Our specification also included maternal height which also may independently influence children height and, if omitted, bias coefficients of education.

12 The result that cluster fixed effects have large effects on the education coefficients is similar to the findings of Desai and Alva (1998) who use first round DHS surveys for 22 countries and approximately 13,000 observations. They find that adding cluster fixed effects reduces the education coefficients by as much as two-thirds, and renders more than half of the previously significant coefficients insignificant. One benefit of our much larger sample is that the education coefficients are much more precisely estimated, and thus do not lose significance even with the addition of cluster fixed effects.

13 In further results not reported for the sake of brevity, we also tested results for different stunting cut-offs, namely severe stunting (HAZ < −3) and mild stunting (HAZ < −1). We found modest changes relative to Regression 4 in Table 3. U*pper secondary* education seems somewhat more important in reducing mild stunting, and primary school somewhat more important in reducing severe stunting. These results are consistent with poorer and more malnourished households having parents who had little access to secondary education. Note that we also estimated results with Probit regressions, but these were very similar to the linear probability model results.

14 Previous research on Egypt—which dominates this sample—has also noted the weak relationship between parental education and undernutrition in this region (El-Kogali & Krafft, 2015).

15 It is not possible, however, to instrument the individual decisions and then to determine their specific impact on child growth. Moreover, as the DHS surveys do not always include the same set of questions, the sample of countries and the sample size varies. Nevertheless, collectively the results in these regressions point to multiple pathways that can mediate the role of education.
